# Risk factors for invasive fungal disease in critically ill adult patients: a systematic review

**DOI:** 10.1186/cc10574

**Published:** 2011-11-29

**Authors:** Hannah Muskett, Jason Shahin, Gavin Eyres, Sheila Harvey, Kathy Rowan, David Harrison

**Affiliations:** 1 Intensive Care National Audit & Research Centre, Tavistock House, Tavistock Square, London, WC1H 9HR, UK; 2 Department of Health Services Research & Policy, Faculty of Public Health & Policy, London School of Hygiene & Tropical Medicine (LSHTM), Keppel Street, London, WC1E 7HT, UK; 3 Department of Medical Statistics, Faculty of Epidemiology and Community Health, London School of Hygiene and Tropical Medicine, Keppel Street, London, WC1E 7HT, UK

## Abstract

**Introduction:**

Over 5,000 cases of invasive *Candida *species infections occur in the United Kingdom each year, and around 40% of these cases occur in critical care units. Invasive fungal disease (IFD) in critically ill patients is associated with increased morbidity and mortality at a cost to both the individual and the National Health Service. In this paper, we report the results of a systematic review performed to identify and summarise the important risk factors derived from published multivariable analyses, risk prediction models and clinical decision rules for IFD in critically ill adult patients to inform the primary data collection for the Fungal Infection Risk Evaluation Study.

**Methods:**

An internet search was performed to identify articles which investigated risk factors, risk prediction models or clinical decisions rules for IFD in critically ill adult patients. Eligible articles were identified in a staged process and were assessed by two investigators independently. The methodological quality of the reporting of the eligible articles was assessed using a set of questions addressing both general and statistical methodologies.

**Results:**

Thirteen articles met the inclusion criteria, of which eight articles examined risk factors, four developed a risk prediction model or clinical decision rule and one evaluated a clinical decision rule. Studies varied in terms of objectives, risk factors, definitions and outcomes. The following risk factors were found in multiple studies to be significantly associated with IFD: surgery, total parenteral nutrition, fungal colonisation, renal replacement therapy, infection and/or sepsis, mechanical ventilation, diabetes, and Acute Physiology and Chronic Health Evaluation II (APACHE II) or APACHE III score. Several other risk factors were also found to be statistically significant in single studies only. Risk factor selection process and modelling strategy also varied across studies, and sample sizes were inadequate for obtaining reliable estimates.

**Conclusions:**

This review shows a number of risk factors to be significantly associated with the development of IFD in critically ill adults. Methodological limitations were identified in the design and conduct of studies in this area, and caution should be used in their interpretation.

## Introduction

In the past, invasive fungal disease (IFD) was more commonly found in patients who were neutropenic, had received a solid organ transplant or had been treated with corticosteroids or cytotoxic agents. Increasingly, IFD is now more likely to occur in nonneutropenic patients in critical care units [1]. The majority of IFD in the critical care setting is due to *Candida *species [2,3]. In 2006, the Health Protection Agency (HPA) estimated that over 5,000 cases of invasive *Candida *species infections occur in the UK each year and that around 40% of these occur in critical care units [4]. An epidemiological survey in six UK sentinel hospitals reported that 45% of *Candida *bloodstream infections occur in the critically ill [5]. IFD in critically ill patients is associated with increased morbidity and mortality at a cost to both the individual and the National Health Service [6,7].

A number of randomised controlled trials (RCTs) have evaluated antifungal prophylaxis in nonneutropenic, critically ill patients, predominantly with either fluconazole [8-12] or ketoconazole [13-16]. Several systematic reviews and meta-analyses of these studies have been performed [17-22]. These reviews reveal that, across the individual studies, patient groups were heterogeneous, ranging from high-risk surgical patients [11,12,16] to those with septic shock [8] or acute respiratory distress syndrome [13,15]. All of the patient groups, however, were at high risk of IFD, with rates in the control arms typically being over 10%. Despite this heterogeneity, the RCTs demonstrated a remarkably homogeneous effect of antifungal prophylaxis on the risk of proven IFD with a suggested reduction in all-cause mortality [17]. The question, therefore, is not whether antifungal prophylaxis is effective, but rather how to select an appropriate group of high-risk patients to receive prophylaxis, as indiscriminate use of antifungal agents is likely to promote drug resistance and drive up cost.

The Fungal Infection Risk Evaluation (FIRE) Study was undertaken with the aim of developing and validating a risk model to identify critically ill nonneutropenic patients at high risk of IFD who would benefit from antifungal prophylaxis (UK Clinical Research Collaboration registered ID number 42) [https://www.icnarc.org/CMS/ArticleDisplay.aspx?ID=8234e564-5902-de11-b27f-0015c5e673e7&root=RESEARCH&categoryID=70422f67-6983-de11-9a46-002264a1a658]). The first step in model development was to prospectively gather data on risk factors for IFD for this patient group. This paper reports the results of a systematic review performed to identify and summarise the important risk factors from published multivariable analyses, risk prediction models and clinical decision rules for IFD in critically ill adult patients to inform the primary data collection in the FIRE Study.

## Materials and methods

An internet search was performed using MEDLINE (1950 to 2008, SilverPlatter WebSPIRS, http://www.ovid.com/site/products/tools/silverplatter/sp_webspirs.jsp; Ovid/Wolters Kluwer Health, New York, NY, USA), Embase (1947 to 2008, http://www.embase.com/; SilverPlatter WebSPIRS) and *CINAHL *(1960 to 2008, EBSCO*host*, http://www.ebscohost.com/cinahl/; EBSCO Publishing, Ipswich, MA, USA) to identify published English-language articles which (1) investigated the predictive value of risk factors for IFD in critically ill adult patients, or (2) developed or evaluated a risk score or risk prediction model for IFD in critically ill adult patients or (3) developed or evaluated a clinical decision rule or patient algorithm for use of antifungal prophylaxis in critically ill adult patients. Three search phrases were combined: 'fungal disease and treatment', 'patient population' and 'risk factors/risk models/clinical rules' (see Additional file 1 for search strategy).

Articles were identified in a staged process whereby titles were initially screened for potential eligibility by a single reviewer (GE). Abstracts and full texts of those potentially eligible were then assessed by two reviewers (HM and JS) independently and were included if the following criteria were met: (1) evaluation of multiple risk factors, a scoring system or a clinical decision rule for IFD in critically ill patients; (2) inclusion of a control group consisting of patients without IFD or any other systemic infection and (3) study of adult humans (age > 18 years). Any disagreements between the reviewers were resolved by a third (DH). Following review of abstracts, we obtained full-text articles for all that were eligible for inclusion. At this point, members of the FIRE Study Steering Group (see Acknowledgements), as clinical experts in the field, were contacted to determine if any relevant articles were missed.

Data were extracted onto standardised data extraction sheets independently by two reviewers (HM and JS; data extraction sheets available on request). The following data were abstracted from each article: study design, method of data collection, clinical setting, population characteristics, method of analysis, risk factors reported, outcome (types and definitions of IFD) and strength of association demonstrated. Data were gathered on the adjusted ORs, 95% CIs, and *P*-values for each of the studied risk factors.

The methodological quality of the reporting of the selected articles was assessed independently by two reviewers (HM and JS) using a set of questions addressing both general and statistical methodologies. Given that no gold standard method exists for the methodological assessment of risk factor studies, the questions were drawn from research from a published quality assessment method for randomised and nonrandomised studies [23], as well as from research on reporting of prognostic models in the oncology field [24,25].

Eight questions assessed the general methodology: study objectives, outcome description, patient characteristics, number of centres recruited, existence of an *a priori *analysis plan, adjustment for known risk factors, rationale behind risk factor inclusion and definition of risk factors. In assessing whether study results were adjusted for known risk factors, the factors considered were severity of illness, length of stay, antibiotics use, receipt of total parenteral nutrition, immunosuppressant use, diabetes, renal dysfunction or renal replacement therapy, central venous catheter use and major surgery. These risk factors were selected based on expert clinical opinion. A study was recorded as adjusting for a majority of the known risk factors if six or more of the nine risk factors were accounted for.

Three questions assessed the statistical methodology: adequacy of sample size, risk factor selection and model strategy choice. Adequacy of sample size was established using the generally held rule of ten events per variable [25]. All risk factors included in the statistical modelling, including those excluded from multivariable modelling following univariable screening, were included in the calculation of events per variable. 'Risk factor selection' referred to how risk factors were entered into the multivariable logistic regression model. The selection process was based on univariable analysis, previous literature or investigator choice, or no selection strategy, whereby all risk factors were entered into the model. 'Model strategy' consisted of either forward selection, backward elimination or no stepwise process, whereby all risk factors were kept in the model. If details on the risk factor selection and model strategy were absent, then they were labelled as unclear.

## Results

**Figure 1:**
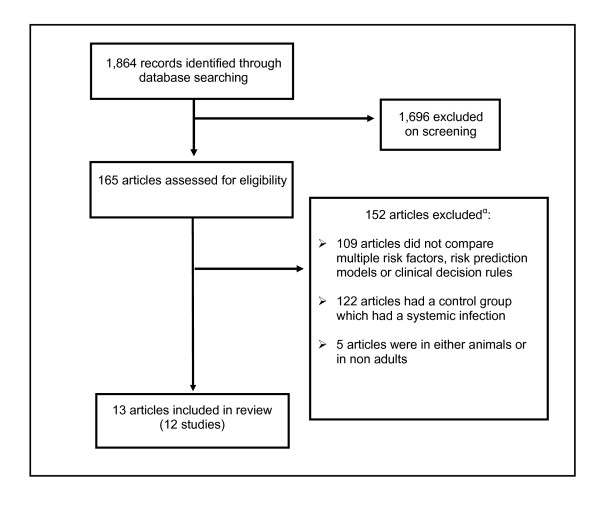
**Article flow through different stages of the review**. α = Articles may have more than one reason for exclusion.

The electronic search identified a total of 1,864 citations (Figure 1). After screening of titles, 165 articles were selected for abstract and full-text review, and 152 of these that were potentially eligible were excluded because they failed to meet the inclusion criteria. Among these, 109 did not assess multiple risk factors, a scoring system and/or a clinical decision rule; 122 articles had a control group which had a systemic infection; and 5 were not adult human studies. Some articles were excluded for multiple reasons. No additional articles were identified by the clinical experts consulted.

The 13 articles that met the inclusion criteria fell into three groups: 8 articles examined risk factors, 4 developed a risk prediction model or clinical decision rule and 1 was an evaluation of a clinical decision rule. Two of the articles utilised data from the same study: the EPCAN Study [26,27]. There were three case-control and nine cohort studies with varying inclusion criteria, including age and length of stay in the critical care unit. The studies were conducted in various countries: Brazil, France, Greece, Spain, Sweden, Switzerland and the USA. Six were based on general critical care patients, and the patients in the rest of the articles were selected from specialised units, including surgical, cardiac and trauma units. Studies varied greatly in terms of outcome definitions. Four studies reported only on *Candida *infections in blood, four used European Organization for Research and Treatment of Cancer/Invasive Fungal Infections Cooperative Group and the National Institute of Allergy and Infectious Diseases Mycoses Study Group (EORTC/MSG) criteria or modifications thereof, and the rest used alternative definitions. Given the heterogeneity of the studies, no meta-analysis was performed. The general characteristics of the selected studies are shown in Table 1.

**Table 1:** Characteristics of selected studies

Study	Study type	Selection criteria	Study design	Number of ICUs	Total patients	Patients with outcomes/cases	Outcome/case definition
Agvald-Ohman *et al*., 2008 [28]	Risk factor analysis	Any multidisciplinary ICU patients LOS ≥ 7 days	Prospective cohort	1	59	10	Blood and/or sterile body site culture positive for *Candida *species
Blumberg *et al*., 2001 [29]	Risk factor analysis	SICU patients LOS > 48 hours	Prospective cohort	6	4,276	42	*Candida *species recovered from culture of blood specimen collected > 48 hours after admission to SICU
Borzotta & Beardsley, 1999 [34]	Risk factor analysis	Trauma ICU patients Cases selected if LOS > 4 days, age > 16 years and any evidence of fungal infection or treatment Controls matched for sex, mechanism of injury, age and Injury Severity Score Controls selected at 2:1 ratio	Case-control	1	656	20	Blood culture positive for yeast infection Yeast from any sterile area Funguria with signs of sepsis and no bacterial pathogen source if > 105 colonies/ml yeast *Candida *growth at two sites with fever and WBC count > 12,000/μl and no bacterial isolates within 48 hours
Chow *et al*., 2008 [30]	Risk factor analysis	Medical or SICU patients Cases selected if positive blood culture for *Candida *species after the first 48 hours of admission to unit Controls matched for study hospital, ICU type and admission date Controls selected at 5:1 ratio	Case-control	2	926	146	At least one blood culture positive for *Candida *species
Ibàñez-Nolla *et al*., 2004 [31]	Risk factor analysis	Any multidisciplinary ICU patients *Candida *species in culture or on histological examination during ICU stay or postmortem Neutrophil count ≥ 500/mm^3^	Prospective cohort	1	145	120	Multifocal candidiasis: simultaneous isolation of *Candida *species in two or more of the following locations: respiratory, digestive or urinary tract or other locations *or* Disseminated candidiasis, yeasts in fluids from sterile sites or histological samples from deep organs or diagnosis of endophthalmitis or candidaemia with negative catheter tip cultures Also, use of EORTC/MSG guidelines
Jordà-Marcos *et al*., 2007 [26]^a^	Risk factor analysis	Any multidisciplinary ICU patients Age > 18 years LOS ≥ 7 days	Prospective cohort	73	1,765	63	At least one blood culture positive for *Candida *species
León *et al*., 2006 [27]^a^	Development of risk prediction model	Multidisciplinary ICU patients Age > 18 years LOS ≥ 7 days Only patients with fungal colonisation included in risk factor analysis/model development	Prospective cohort	73	1,699	97	Candidaemia Candidal endophthalmitis in a patient with clinical sepsis *Candida *species from sterile sites Histologically documented candidiasis
Michalopoulos *et al*., 2003 [36]	Risk factor analysis	Cardiothoracic ICU patients Cases selected if at least one blood culture positive for *Candida *species detected Controls matched for admission date, gender, BMI, sedatives, CPB technique and cardioplegia type Controls selected at 4:1 ratio	Case-control	1	150	30	At least one blood culture positive for *Candida *species
McKinnon *et al*., 2001 [32]	Risk factor analysis	SICU patients LOS ≥ 5 days Age > 18 years	Prospective cohort	3	301	27	Colonisation of two or more sites or candidaemia
Ostrosky-Zeichner *et al*., 2007 [37]	Development of clinical decision rule	Multidisciplinary ICU patients Age ≥ 19 years LOS ≥ 4 days No evidence of invasive candidiasis or systemic antifungal use in week prior to ICU admission through first 3 days of admission	Retrospective cohort	12	2,890	88	EORTC/MSG criteria
Paphitou *et al*., 2005 [35]	Development of clinical decision rule	SICU patients LOS ≥ 4 days	Retrospective cohort	1	327	36	Based on proven, probable or possible cases Criteria modelled on EORTC/MSG criteria
Piarroux *et al*., 2004 [38]	Evaluation of clinical decision rule	SICU patients LOS ≥ 5 days Excluded liver transplants	Prospective and retrospective cohorts	1	933	50	EORTC/MSG criteria
Pittet *et al*., 1994 [33]	Risk factor analysis and development of clinical decision rule	SICU/neonatal ICU patients *Candida *colonisation in three or more samples on two consecutive days	Prospective cohort	2	29	11	Candidaemia *or* One blood culture with one histologically documented invasive candidiasis *or* Ophthalmic examination consistent with candidal endophthalmitis *or* At least two blood cultures taken at different times *or* One peripheral blood culture and one central line blood culture showing identical *Candida *species *or* Severe nonbloodstream *Candida *species infection *Candida *species in normally sterile site and at least one of the following: Fever or hypothermia Unexplained, prolonged hypotension No response to adequate antibiotic treatment for a suspected bacterial infection

BMI = body mass index; CPB = cardiopulmonary bypass; EORTC/MSG = European Organization for Research and Treatment of Cancer/Invasive Fungal Infections Cooperative Group and the National Institute of Allergy and Infectious Diseases Mycoses Study Group; LOS = length of stay; SICU = surgical ICU. ^a^Articles from the EPCAN Study.

### Analysis of risk factors

The risk factors examined varied between the studies. Table 2 reports all the risk factors that were identified as statistically significantly associated with IFD in one or more of the 10 studies (11 articles) that carried out a multivariable analysis. Table 3 reports all significant risk factors that were examined, and the number of studies in which these were associated with IFD, on univariable and multivariable analyses. Candidate risk factors, in descending order of the number of studies in which the risk factor was significantly associated on multivariable analysis, are described below.

**Table 2:** Risk factors and adjusted effect estimates

Risk factors	Studies	OR (95% CI)	*P*-values
Surgery			
General abdominal surgery	Agvald-Ohman *et al*., 2008 [28]	60.7 (7.3 to infinity)	0.0013
Any surgery	Blumberg *et al*., 2001 [29]	7.3 (1 to 53.8)	0.05
Elective surgery	Jordà-Marcos *et al*., 2007 [26]^a^	2.75 (1.17 to 6.45)	0.02
Surgery on ICU admission	León *et al*., 2006 [27]^a^	2.71 (1.45 to 5.06)	< 0.001
Gastrointestinal procedure	Chow *et al*., 2008 [30]	2.24 (1.49 to 3.38)^β^	< 0.001^β^
Major pre-ICU operation	Chow *et al*., 2008 [30]	2.12 (1.14 to 3.97)^β^	0.02^β^
Major operation during ICU stay	Chow *et al*., 2008 [30]	1.26^α^	0.04^α^
Multiple surgical procedures	McKinnon *et al*., 2001 [32]	NR	≤ 0.05
Total parenteral nutrition			
Total parenteral nutrition duration/days at risk	Chow *et al*., 2008 [30]	11 (5.52 to 21.7)^α^	< 0.01^α^
		2.87 (1.4 to 5.9)^β^	< 0.01^β^
Total parenteral nutrition	Jordà-Marcos *et al*., 2007 [26]^a^	3.89 (1.73 to 8.78)	0.001
Total parenteral nutrition	Blumberg *et al*., 2001 [29]	3.6 (1.8 to 7.5)	< 0.001
Total parenteral nutrition	León *et al*., 2006 [27]^a^	2.48 (1.16 to 5.31)	< 0.001
Total parenteral nutrition	Borzotta & Beardsley, 1999 [34]	NR	< 0.001
Fungal Colonisation			
Digestive focus	Ibàñez-Nolla *et al*., 2004 [31]	20.24 (6.11 to 67.03)	< 0.001
Colonisation Index ≥ 0.5	Agvald-Ohman *et al*., 2008 [28]	19.1 (2.38 to 435)	0.017
Non-*Candida albicans *at screening	Ibàñez-Nolla *et al*., 2004 [31]	11.68 (1.93 to 70.63)	0.007
Respiratory focus	Ibàñez-Nolla *et al*., 2004 [31]	6.55 (1.25 to 34.3)	0.026
*Candida *colonisation	Jordà-Marcos *et al*., 2007 [26]^a^	4.12 (1.82 to 9.33)	0.001
*Candida *colonisation	León *et al*., 2006 [27]^a^	3.04 (1.45 to 6.39)	< 0.001
*Candida *species corrected colonisation index	Pittet *et al*., 1994 [33]	4.01 (2.16 to 7.45)	< 0.001
Renal replacement therapy			
Haemodialysis duration/days at risk	Chow *et al*., 2008 [30]	3.84 (1.75 to 8.4)^α^	< 0.001^α^
		6.2 (2.67 to 14.4)^β^	< 0.0001^β^
New-onset haemodialysis	Paphitou *et al*., 2005 [35]	5.4 (2.5 to 11.8)	0.029
Haemofiltration	Jordà-Marcos *et al*., 2007 [26]^a^	1.96 (1.06 to 3.62)	0.032
Infection/sepsis			
Hospital acquired	Michalopoulos *et al*., 2003 [36]	9.4 (2.5 to 48.3)	< 0.001
Severe sepsis	León *et al*., 2006 [27]^a^	7.68 (4.14 to 14.22)	< 0.001
Enteric bacteraemia	Chow *et al*., 2008 [30]	3.45 (1.38 to 8.63)^α^	< 0.01^α^
		3.43 (1.39 to 8.48)^β^	< 0.01^β^
Mechanical ventilation			
Mechanical ventilation > 10 days	Michalopoulos *et al*., 2003 [36]	28.2 (3.6 to 119.5)	< 0.001
Mechanical ventilation after day 3	McKinnon *et al*., 2001 [32]	NR	≤ 0.05
Diabetes			
Diabetes	Paphitou *et al*., 2005 [35]	2.8 (1.6 to 4.7)	0.053
Diabetes	Michalopoulos *et al*., 2003 [36]	2.4 (1.3 to 13.5)	< 0.01
APACHE II or APACHE III score			
APACHE II score	Pittet *et al*., 1994 [33]	1.03 (1.01 to 1.05)	0.007
APACHE III score	Ibàñez-Nolla *et al*., 2004 [31]	1.03 (1.00 to 1.06)	0.004
Cardiopulmonary bypass time > 120 min	Michalopoulos *et al*., 2003 [36]	8.1 (2.9 to 23.6)	< 0.01
Acute renal failure	Blumberg *et al*., 2001 [29]	4.2 (2.1 to 8.3)	< 0.001
Broad spectrum antibiotics	Paphitou *et al*., 2005 [35]	3.0(1.8 to 5.0)	0.028
Red blood cell transfusion	Chow *et al*., 2008 [30]	1.97 (0.98 to 3.99)^α^	0.06^α^
		2.72 (1.33 to 5.58)^β^	< 0.01^β^
Antifungal medication	Blumberg *et al*., 2001 [29]	0.3 (0.1 to 0.6)	< 0.001
Central venous catheters	McKinnon *et al*., 2001 [32]	NR	≤ 0.05
Diarrhoea	McKinnon *et al*., 2001 [32]	NR	≤ 0.05
Peripheral catheter use	McKinnon *et al*., 2001 [32]	NR	≤ 0.05

APACHE = Acute Physiology and Chronic Health Evaluation; CPB = cardiopulmonary bypass; NR = not reported. α = OR for outcomes in *Candida albicans*; β = OR for outcomes in *Candida *non-*albicans*. ^a^Data combined from both articles from the EPCAN Study.

**Table 3:** Comparison of studies for risk factors associated with invasive fungal disease

Risk factors	Studies examining risk factors (*n*)	Studies where risk factor was significantly associated with IFD on univariable analysis (*n*)	Studies where risk factor was significantly associated with IFD on multivariable analysis (*n*)
Surgery	7	5	5
Total parenteral nutrition	6	6	4
Fungal colonisation	5	4	4
Renal replacement therapy	7	5	3
Infection/sepsis	5	3	3
Mechanical ventilation	5	2	2
Diabetes	4	3	2
APACHE II or APACHE III score	8	2	2
Central venous catheters	7	4	1
Broad-spectrum antibiotics	8	5	1
CPB > 120 minutes	1	1	1
Red blood cell transfusions	3	3	1
Antifungal medication	4	2	1
Acute renal failure	2	1	1
Diarrhoea	1	1	1
Peripheral catheter	1	1	1

APACHE = Acute Physiology and Chronic Health Evaluation; CPB = cardiopulmonary bypass; IFD = invasive fungal disease.

#### Surgery

Seven studies [26-33] examined the association between surgery and IFD. The type and timing of surgery varied across the studies, with two [28,30] looking at abdominal surgery and the others looking at any surgical procedure. Five of the seven studies [26-30,32] reported a significant association between surgery and IFD on both univariable and multivariable analyses.

#### Total parenteral nutrition

Six of the twelve studies [26,27,29,30,32,34,35] assessed total parenteral nutrition as a risk factor, and all found a significant association with IFD on univariable analysis. Of the six studies, four [26,27,29,30,34] also found a significant association on multivariable analysis.

#### Fungal colonisation

Five studies [26-29,31,33] examined the association between fungal colonisation and IFD. Four of the five studies [26-28,31,33] reported an association on both univariable and multivariable analyses. The sites of fungal colonisation examined and modelling approaches varied across the studies.

#### Renal replacement therapy

Seven studies [26,27,30,32-36] examined renal replacement therapy as a risk factor for IFD, of which five [26,27,30,32,34,35] found a significant association on univariable analysis. Three studies [26,30,35] demonstrated a significant association on multivariable analysis. Only one of the two EPCAN articles demonstrated a significant result on multivariable analysis. The type and exposure time to dialysis varied across the studies. Some looked at preadmission dialysis, and others examined haemofiltration in the unit.

#### Infection and sepsis

Five studies [27,29,30,33,36] examined the relationship between infection and sepsis and IFD, three [27,30,36] of which demonstrated an association on multivariable analysis. The source and site of infection varied across the studies. One examined bacterial infection and bacteraemia without specifying type and source of infection [36]. Another examined enteric bacteraemia, which included *Enterococcus, Bacteroides *and other Gram-negative bacilli bloodstream infections [30]. One demonstrated an association with severe sepsis, although the infection source was not specified [27].

#### Mechanical ventilation

Five studies [26,27,29,32,34,36] examined the association between receipt of mechanical ventilation and IFD. Two of the five studies [32,36] reported a significant association on multivariable analysis. Both timing and duration of mechanical ventilation varied across the studies, with one study examining mechanical ventilation on day 3 of critical care unit admission [32] and the other on day 10 [36].

#### Diabetes

Four studies [27,28,35,36] examined whether a medical history of diabetes mellitus was a risk factor for IFD. Two of the four studies [35,36] demonstrated a significant association on both univariable and multivariable analyses.

#### APACHE II or APACHE III score

Eight studies [26-29,31,33-35] examined whether either the Acute Physiology and Chronic Health Evaluation II (APACHE II) score or APACHE III score was a risk factor for IFD. Two [31,33] of the eight studies demonstrated a significant association on both univariable and multivariable analyses.

#### Other risk factors

A number of other risk factors were identified as being significantly associated with IFD on multivariable analysis in single studies. These included cardiopulmonary bypass time, acute renal failure, broad-spectrum antibiotic use, red blood cell transfusions, antifungal medication use, central venous catheter use, diarrhoea and peripheral catheter use (Table 2). Of note, two studies [26,37] examined the association between neutropenia and IFD, neither of which demonstrated a significant association. Similarly, none of the five studies [27,28,33-35] looking at immunosuppressant use demonstrated an association with IFD.

### Risk prediction models and clinical decision rules

Four of the studies developed a risk prediction model or clinical decision rule (Table 4), and one evaluated a clinical decision rule for IFD, all in the critical care setting. León *et al*. [27] developed and validated a risk prediction model from which they derived a bedside scoring system to inform early antifungal treatment in nonneutropenic critically ill patients. The study comprised a prospective cohort of 1,699 patients, of whom 980 with colonisation or infection were included in the model development with 97 IFDs. Multifocal *Candida *colonisation, surgery directly prior to critical care unit admission, severe sepsis and total parenteral nutrition were included in the final risk prediction model. The optimal score from the model gave a sensitivity of 81% and a specificity of 74%.

**Table 4:** Studies developing risk models or clinical decision rules

Study	Development	Validation	Models/rules	AUC	Sensitivity	Specificity	PPV	NPV
León *et al*., 2006 [27]	Risk factors significant (*P *< 0.05) on univariable analysis of full sample included in multivariable logistic regression model fitted in 65% development sample Final model chosen by backward elimination; stopping criterion unclear Simplified to bedside score by rounding coefficients.	ROC and sensitivity/specificity at cut-off values in 35% validation sample	0.908 × (total parenteral nutrition) + 0.997 × (surgery) + 1.112 × (multifocal *Candida *species colonisation) + 2.038 × (severe sepsis)	0.847 (0.800 to 0.894)	NA	NA	NA	NA
			1 × (total parenteral nutrition) + 1 × (surgery) + 1 × (multifocal *Candida *species colonisation) + 2 × (severe sepsis)	NR	NA	NA	NA	NA
			Bedside score ≥ 3, or, equivalently, severe sepsis plus at least one other risk factor *or *all three other risk factors	NA	81%	74%	NR	NR
Ostrosky-Zeichner *et al*., 2007 [37]	All rule development in 75% development sample Univariable analysis of risk factors Clinical decision rules constructed for 'all possible combinations of risk factors and time points' in 'several different formats (with different weights for the risk factors)' 'Best' rules selected on sensitivity, PPV, PPV/(1-NPV), and proportion of patients identified as high risk in development sample	χ^2 ^test of association, sensitivity, specificity, PPV and NPV in 25% validation sample	Any antibiotic days 1 to 3 *and *CVC days 1 to 3	NA	89%	38%	4%	99%
			Any antibiotic days 1 to 3) *and *CVC days 1 to 3 *and *at least one of the following: Any surgery days -7 to 0 Immunosuppressive use days -7 to 0 Pancreatitis days -7 to 0 TPN day 1-3; dialysis day 1-3 Steroid use days -7 to 3	NA	66%	69%	6%	98%
			Any antibiotic days 1 to 3 *or *CVC days 1 to 3 *and *at least two of the following: Any surgery days -7 to 0 Immunosuppressive use days -7 to 0 Pancreatitis days -7 to 0 TPN days 1 to 3 Dialysis days 1 to 3 Steroid use days -7 to 3	NA	34%	90%	9%	97%
Paphitou *et al*., 2005 [35]	All rule development in full sample Univariable analysis of risk factors; unclear whether used to select factors for multivariable model Multivariable logistic regression model with stepwise procedure; unclear whether forward selection or backward elimination and unclear stopping criterion Clinical decision rules constructed 'using a combination of inspection of the data and results of the multivariable analysis'	Sensitivity and PPV in full sample NNT assuming 50% relative reduction in IFD associated with treatment Cost to prevent one case assuming prophylaxis $100/day	At least one of the following: Diabetes mellitus TPN days -7 to 0 New-onset haemodialysis days -7 to 3	NA	39%	NR	17% to 26%	NR
			At least one of the following: Diabetes mellitus TPN days -7 to 0 New-onset haemodialysis days -7 to 3 Broad-spectrum antibiotics days -7 to 3	NA	78% to 83%	NR	11% to 17%	NR
			At least one of the following: Diabetes mellitus TPN days -7 to 0 New-onset haemodialysis days -7 to 3 *and* Broad-spectrum antibiotics days -7 to 3	NA	30% to 33%	NR	20% to 34%	NR
Pittet *et al*., 1994 [33]	All rule development in full sample Clinical decision rules constructed from colonisation parameters only (number of sites, colonisation index, corrected colonisation index derived *post hoc*); methods unclear Risk factors with *P *< 0.15 on univariable analysis included in multivariable logistic regression model Only those with *P *< 0.05 in multivariable model reported; unclear if stepwise procedure used	Clinical decision rules validated by sensitivity, specificity, PPV and NPV in full sample	Colonisation at two or more sites	NA	100%	22%	44%	100%
			Colonisation at three or more sites	NA	73%	56%	50%	77%
			Colonisation at four or more sites	NA	45%	72%	50%	68%
			*Candida *colonisation index ≥ 0.5	NA	100%	69%	66%	100%
			*Candida *corrected colonisation index ≥ 0.4	NA	100%	100%	100%	100%

AUC = area under the receiver operating characteristic curve; CVC = central venous catheter; IFD = invasive fungal disease; NA = not applicable; NNT = number needed to treat to prevent one case; NPV = negative predictive value; NR = not reported; PPV = positive predictive value; ROC = receiver operating characteristic curve; TPN = total parenteral nutrition.

Ostrosky-Zeichner *et al*. [37] developed a number of clinical decision rules for IFD in the critical care setting. Their study was a retrospective chart review of 2,890 patients from 12 participating centres with 88 cases of IFD. Several clinical decision rules, with varying combinations of risk factors, were developed and tested. The best-performing rule consisted of the following risk factors: any systemic antibiotic, presence of a central venous catheter. In addition, their rule included at least two of the following risk factors: total parenteral nutrition, any dialysis, any major surgery, pancreatitis and use of steroids or other immunosuppressants. The sensitivity and specificity of the model were 34% and 90%, respectively.

Paphitou *et al*. [35] developed and validated a number of clinical decision rules from a single-centre, retrospective cohort study of 327 critically ill patients comprising 9 cases of proven IFD and 27 probable or possible cases. Several combinations of risk factors were evaluated, of which any combination of diabetes mellitus, new-onset haemodialysis, use of total parenteral nutrition or receipt of broad-spectrum antibiotics was considered the most useful. The model had a sensitivity of 78% to 83% and specificity of approximately 50%.

Pittet *et al*. [33] developed a number of clinical decision rules based on intensity of *Candida *colonisation from a single-centre, prospective cohort study of 29 critically ill patients with significant *Candida *colonisation, of whom 11 had severe *Candida *infection. The best-performing rule, developed *post hoc *to give perfect discrimination in the small data set, was a *Candida *corrected colonisation index (ratio of highly positive fungal screening samples to the total number of samples) of 0.4 or more.

Piarroux *et al*. [38] evaluated the clinical decision rule developed by Pittet *et al*. [33] whereby patients admitted to a single surgical intensive care unit were screened for fungal colonisation and preemptively treated with fluconazole if the *Candida *corrected colonisation index was 0.4 or more. On the basis of a review of same-centre historical controls from a time period prior to prophylaxis, a reduction of unit-acquired IFD from 2.2% to 0% (*P *< 0.001) was reported.

### Reporting of methodological assessment

The included studies varied with respect to their methodological quality (Tables 5 and 6). All 12 studies reported objectives, main outcomes and characteristics of the selected study patients (Table 5). The majority of the studies were carried out in at least two critical care units. The analysis was defined *a priori *in ten (83%) of the twelve studies, and the majority of known risk factors were accounted for in ten (91%) of the eleven studies in which multivariable analyses were conducted. The study by Piarroux *et al*. [38] evaluated a clinical decision rule and therefore did not carry out a risk factor analysis. Risk factors were poorly defined in over half of the studies, and a rationale for inclusion was missing in over two-thirds of the studies.

**Table 5:** Methodology and reporting assessment: general assessment

Study	Is study objective clearly described?	Are main outcomes measured clearly described?	Are patient characteristics clearly described?	Was study performed in multiple centres (more than two)?	Was analysis defined *a priori*?	Did analysis account for majority of known risk factors?	Was rationale behind inclusion of risk factors included?	Were risk factors clearly defined?
Agvald-Ohman *et al*., 2008 [28]	✓	✓	✓	x	x	x	x	x
Blumberg *et al*., 2001 [29]	✓	✓	✓	✓	✓	✓	x	x
Borzotta & Beardsley, 1999 [34]	✓	✓	✓	✓	✓	✓	x	✓
Chow *et al*., 2008 [30]	✓	✓	✓	✓	✓	✓	x	x
Ibàñez-Nolla *et al*., 2004 [31]	✓	✓	✓	x	✓	✓	x	x
Jordà-Marcos *et al*., 2007 [26]	✓	✓	✓	✓	✓	✓	x	✓
León *et al*., 2006 [27]	✓	✓	✓	✓	✓	✓	x	✓
Michalopoulos *et al*., 2003 [36]	✓	✓	✓	✓	✓	✓	✓	✓
McKinnon *et al*., 2001 [32]	✓	✓	✓	✓	✓	✓	✓	✓
Ostrosky-Zeichner *et al*., 2007 [37]	✓	✓	✓	✓	x	✓	x	x
Paphitou *et al*., 2005 [35]	✓	✓	✓	x	✓	✓	✓	x
Piarroux *et al*., 2004 [38]	✓	✓	✓	x	✓	n/a	n/a	n/a
Pittet *et al*., 1994 [33]	✓	✓	✓	x	x	✓	x	✓

n/a = not applicable (evaluation of clinical decision rule, no risk factor analysis).

**Table 6:** Methodology and reporting assessment: statistical assessment

	Power				Risk factor selection^α^				Statistical model^α^			
	
Article	Events	Variables	EPV	Were there ≥ 10 events per risk factor?	All Candidate risk factors used	Risk factors chosen based on previous literature/investigator choice	Risk factors chosen based on univariable analysis	Unclear	No selection: all variables kept in model	Backward elimination	Forward selection	Unclear
Agvald-Ohman *et al*., 2008 [28]	10	> 10	< 1	x				✓				✓
Blumberg *et al*., 2001 [29]	42	49	0.9	x				✓		✓		
Borzotta & Beardsley, 1999 [34]	20	> 21	< 1	x			✓			✓		
Chow *et al*., 2008 [30]	67^a^ 79^b^	35	1.9^a^ 2.3^b^	x			✓					✓
Ibàñez-Nolla *et al*., 2004 [31]	120	30	< 4	x				✓				✓
Jordà-Marcos *et al*., 2007 [26]	63	15	4.2	x			✓				✓	
León *et al*., 2006 [27]	97	22	4.4	x			✓			✓		
Michalopoulos *et al*., 2003 [36]	30	29	1.0	x				✓			✓	
McKinnon *et al*., 2001 [32]	27	23	1.2	x			✓					✓
Ostrosky-Zeichner *et al*., 2007 [37]	88	27	3.3	x				✓			✓	
Paphitou *et al*., 2005 [35]	36	> 49	< 0.8	x				✓				✓
Piarroux *et al*., 2004 [38]	50	n/a	n/a	n/a	n/a	n/a	n/a	n/a	n/a	n/a	n/a	n/a
Pittet *et al*., 1994 [33]	11	9	1.2	x			✓					✓

EPV = events per variable; ^a^events/EPV for outcomes in *Candida albicans*; ^b^events/EPV for outcomes in *Candida *non-*albicans*; α = most appropriate of the four options selected in each case; n/a = not applicable (evaluation of clinical decision rule, no risk factor analysis).

Reporting of the statistical modelling was generally poor (Table 6), and it was usually impossible to determine exactly the number of variables that were considered as candidate predictors in each paper. Reported methods often stated, for example, 'Risk factors examined included...', but it was not clear whether the subsequent list was exhaustive, and risk factors could often be determined only from those reported in the results, which in some cases were only those selected by a modelling process or only those that were statistically significant. The numbers of risk factors reported in Table 6 are therefore approximate and, in many cases, a minimum. Some studies split data into development and validation samples, but did not report how many of the events were in the development sample. However, even upon assessing the models on the minimum number of variables included as indicated by the report and the number of events in the full sample (and therefore the maximum events per variable), all of the papers had a strong likelihood of presenting results that were overfitted to the data. Taking into account all variables considered in the statistical modelling (including those screened out on univariable analysis), the largest studies had around four events per variable, and a number of studies had examined at least as many risk factors as there were events in the data set, giving values of one event per variable or less. No studies reached the predefined threshold of ten events per variable. Roughly half of the studies based their decision regarding which risk factors to include in their multivariable analysis on univariable analysis, whereas the reporting in the remaining studies was insufficient to determine risk factor selection. In terms of modelling strategies, one-third of the studies used a forward selection process, one-third used a backward elimination process, and for the remaining third, it was unclear from the reporting which modelling strategy was used.

## Discussion

Thirteen articles were identified which investigated risk factors, risk prediction models or clinical decisions rules for invasive fungal disease in critically ill adult patients. Of these 13, 8 articles examined risk factors specifically, 4 developed risk prediction models or clinical decision rules and 1 evaluated a clinical decision rule.

In this systematic review, the following risk factors were found in multiple studies to be significantly associated with IFD: surgery, total parenteral nutrition, fungal colonisation, renal replacement therapy, infection and sepsis, mechanical ventilation, diabetes and APACHE II or APACHE III score. Cardiopulmonary bypass time, acute renal failure, broad-spectrum antibiotics, red blood cell transfusion, antifungal medication, central venous catheters, diarrhoea and peripheral catheter use were also found to be statistically significant, but each in single studies only. The risk prediction model and clinical decision rule studies employed all of the risk factors found to be significant in multiple studies reported above, apart from mechanical ventilation, and in addition included pancreatitis and immunosuppressant use. Risk factor definitions varied across studies, with many studies offering no definition at all. Risk factor selection process and modelling strategy also varied across studies, and no studies had an adequate sample size for the multivariable analyses. None of the selected studies described the degree of missing data or of how missing data would be handled in the analysis. Some reported numbers of patients included in each model, but reasons for exclusion were unclear.

The risk prediction models and clinical decision rules identified in this review have a number of factors that limit their usefulness for guiding early decision-making regarding antifungal prophylaxis. First, with regard to the patient populations studied, models and rules were developed and evaluated using data from patients whose length of stay in the critical care unit was four [35,37], five [38] or seven days [27]. This would have helped to identify high-risk populations; however, the performance of these models and rules, if applied at an earlier time point in the critical care stay, cannot be determined. Some models and rules were developed on the basis of patients with *Candida *colonisation only [27,33], and consequently they could be used only to guide empiric therapy and not true prophylaxis. Second, with regard to the statistical modelling, models are likely to be overfitted because of the small number of events used for model development. Stepwise selection of risk factors is likely to have resulted in model coefficients that were too large and measures of model performance that were optimistic [39]. Despite having been developed in higher-risk populations identified by longer ICU stays, the specificity of the rules was generally low and hence their use to guide treatment could result in overuse of antifungal drugs, with costs both financial and in terms of increased resistance. No studies have adequately addressed the cost-effectiveness of using clinical decision rules to guide delivery of antifungal therapy. The only study that gave any consideration to costs was that by Paphitou *et al*. [35], who estimated the number needed to treat and associated cost to prevent one case of IFD assuming a relative risk of 0.5 and a cost of $100/day for antifungal prophylaxis. The most promising rule on these criteria had a number needed to treat of six to ten and an associated cost of $12,000 to $21,000 per case prevented.

It is difficult to compare the performance of clinical decision rules across the different studies, owing to the variation in inclusion criteria, outcome definitions and prevalence of the outcome. However, it should be noted that the performance measures are likely to be favourably biased because of overfitting to the data and lack of external validation. Since the end date of our systematic review, three studies have been published validating risk prediction models or clinical decision rules identified in this review. León *et al*. validated their risk prediction model, the Candida Score [27], among a new prospective cohort of 892 admissions with *Candida *colonisation with a stay of at least 7 days in one of 36 multidisciplinary ICUs in Spain, Argentina and France [40]. As expected, the performance of the score was not as good in the validation sample with an area under the receiver operating characteristic curve of 0.77 compared with 0.85 in the development data. Based on a cut-off of a score of 3 or more, the sensitivity was 78% (81% in development data), the specificity was 66% (74% in development data) and positive and negative predictive values were 14% and 98%, respectively (not reported in development data). Playford *et al*. [41] validated four clinical decision rules, the best rule from Ostrosky-Zeichner *et al*. [37] and a subsequent revision of this published in abstract form, and the two best rules from Pittet *et al*. [33], in a prospective cohort of 615 patients admitted for at least 72 hours to 4 multidisciplinary ICUs in Australia. Performance of the clinical prediction rules was worse than in the development data sets, and the authors recommended that to identify a sufficiently high risk population to consider for antifungal treatment would require a combination of the clinical risk factors from Ostrosky-Zeichner *et al*. [37], together with measures of colonisation from Pittet *et al*. [33]. Most recently, Hermsen *et al*. [42] set out to validate the clinical decision rules of Paphitou *et al*. [35] and Ostrosky-Zeichner *et al*. [37] in a case-control study of 88 cases and 264 matched controls staying at least 4 days in a single multidisciplinary ICU in the United States. Rather than validate the rules as published, Hermsen *et al*. fitted new conditional logistic regression models using the risk factors from these rules, rendering their results incomparable with the original publications. It is worth noting, however, that a number of the risk factors included in the rules (surgery, pancreatitis, haemodialysis and diabetes) were not found to be significantly associated with risk of IFD.

This review is the first to systematically evaluate and assess the quality of the literature on risk factors for IFD. Rigorous search methods and a tailored quality assessment tool were combined to produce a high-quality systematic review. As search strategies are designed for identifying randomised controlled trials rather than risk factor studies, a comprehensive search strategy including multiple medical subject heading terms and keywords describing risk, risk prediction models and clinical decision rules were employed. Furthermore, abstracts and full-text articles were reviewed, and data extracted, by two investigators independently to ensure that all relevant articles and data were captured. There is currently no validated gold standard or single recommended instrument for methodological assessment of risk factor studies, so a combined methodological assessment was developed for this review and tailored to assess the specific areas of risk factor studies which were considered to be important.

One limitation of our review is that the heterogeneity of the selected articles precluded any meta-analysis. Study objectives differed between the studies, with some assessing a specific clinical decision rule and some examining a range of risk factors. The ways in which the risk factors and outcomes were defined also differed, and different inclusion criteria were imposed across the studies, making combination of results inappropriate. The existence of publication bias is always a possibility in systematic reviews, but many risk factors were shown to be nonsignificant on multivariable analysis, indicating that negative as well as positive results were represented in the studies. In the univariable analysis however, it was difficult to identify which risk factors were nonsignificant, as the full list of factors examined was not always made clear.

## Conclusion

In conclusion, this review has shown a number of risk factors to be significantly associated with the development of IFD in critically ill adults. However, this review has highlighted numerous methodological limitations in the design and conduct of studies in this area, and as such it is suggested that caution should be used in their interpretation. These results form an important underpinning for a large, publicly funded, prospective cohort study, the FIRE Study, which aims to develop and validate a risk model to effectively target antifungal prophylaxis to critically ill nonneutropenic patients at high risk of IFD. The first step in model creation was to prospectively gather data on risk factors and outcomes for this patient group, which was guided by the results of this review.

## Key messages

◆ IFD in critically ill patients is associated with increased morbidity and mortality at a cost to both the individual and the healthcare system.

◆ Thirteen articles which investigated risk factors, risk prediction models or clinical decisions rules for IFD in critically ill adult patients were identified.

◆ Multiple risk factors were found to be associated with IFD on univariable and multivariable analyses.

◆ Studies varied in terms of objectives, risk factors, definitions and outcomes.

## Abbreviations

APACHE: Acute Physiology and Chronic Health Evaluation; CPB: cardiopulmonary bypass; EORTC/MSG: European Organization for Research and Treatment of Cancer/Invasive Fungal Infections Cooperative Group and the National Institute of Allergy and Infectious Diseases Mycoses Study Group; FIRE: Fungal Infection Risk Evaluation; IFD: invasive fungal disease; LOS: length of stay; MeSH: medical subject heading; RCT: randomised control trial; SICU: surgical ICU.

## Competing interests

The authors declare that they have no competing interests.

## Authors' contributions

HM, JS and GE participated in the design of the study, data collection and analysis and contributed to drafting the manuscript. SH participated in data analysis and contributed to drafting the manuscript. KR and DH participated in the design of the study and data analysis and contributed to drafting the manuscript. All authors read and approved the final manuscript.

## Acknowledgements and funding

The authors thank the FIRE Study Steering Group: Dr Ronan McMullan, Dr Bernard Riley, Dr Thomas Stambach, Dr Rosemary Barnes, Dr Jonathan Edgeworth, Dr Richard Grieve, Dr Mark Jit, Prof Christopher Kibbler and Dr Neil Soni. This project was funded by the NHS National Institute of Health Research (NIHR) Health Technology Assessment programme and will be published in full in the Health Technology Assessment (HTA) journal series. Visit the HTA programme website for more details http://www.hta.ac.uk/1720. The views and opinions expressed therein are those of the authors and do not necessarily reflect those of the Department of Health.

## Supplementary Material

Additional file 1 **Appendix 1 Search strategy**. Click here for file
