# Supplementary material for: Risk factors for invasive fungal disease in critically ill adult patients: a systematic review
Source: Crit Care. 2011 Nov 29;15(6):R287. doi: 10.1186/cc10574 (PMC3388661; doi:10.1186/cc10574)
Supplement: Additional file 1 — Appendix 1 Search strategy. [file cc10574-S1.DOCX]

# Appendix 1: Search Strategy

| 1 | . | Mycoses in MESH |
| --- | --- | --- |
| 2 | . | Antifungal Agents in MESH |
| 3 | . | fung* in AB/TI/KW |
| 4 | . | Candida in AB/TI/KW |
| 5 | . | candidemia in AB/TI/KW |
| 6 | . | candidaemia in AB/TI/KW |
| 7 | . | candidiasis in AB/TI/KW |
| 8 | . | Candidal in AB/TI/KW |
| 9 | . | fluconazole in AB/TI/KW |
| 10 | . | Diflucan in AB/TI/KW |
| 11 | . | itraconazole in AB/TI/KW |
| 12 | . | sporanox in AB/TI/KW |
| 13 | . | ketocanazole in AB/TI/KW |
| 14 | . | nizoral in AB/TI/KW |
| 15 | . | voriconazole in AB/TI/KW |
| 16 | . | amphotericin in AB/TI/KW |
| 17 | . | ambisome in AB/TI/KW |
| 18 | . | amphotec in AB/TI/KW |
| 19 | . | abelcet in AB/TI/KW |
| 20 | . | flucytosine in AB/TI/KW |
| 21 | . | Nystatin in AB/TI/KW |
| 22 | . | miconazole in AB/TI/KW |
| 23 | . | echinocandin* in AB/TI/KW |
| 24 | . | caspofungin in AB/TI/KW |
| 25 | . | (select* NEAR decontam*) in AB/TI/KW |
| 26 | . | OR/1-25 |
| 27 | . | Intensive Care Units in MESH |
| 28 | . | Critical Care in MESH |
| 29 | . | intensive care in AB/TI/KW |
| 30 | . | critical care in AB/TI/KW |
| 31 | . | critical illness in AB/TI/KW |
| 32 | . | critically ill in AB/TI/KW |
| 33 | . | OR/27-32 |
| 34 | . | 26 and 33 |
| 35 | . | Risk in MESH |
| 36 | . | Models, Statistical in MESH |
| 37 | . | Regression Analysis in MESH |
| 38 | . | Sensitivity and Specificity in MESH |
| 39 | . | Survival Analysis in MESH |
| 40 | . | Operations Research in MESH |
| 41 | . | Decision Support Techniques in MESH |
| 42 | . | Clinical Protocols in MESH |
| 43 | . | Practice Guidelines in MESH |
| 44 | . | Patient Selection in MESH |
| 45 | . | risk* in AB/TI/KW |
| 46 | . | predict* in AB/TI/KW |
| 47 | . | model* in AB/TI/KW |
| 48 | . | rule* in AB/TI/KW |
| 49 | . | ((decision OR algorithm) NEAR (clinical or treatment or prophyla*)) in AB/TI/KW |
| 50 | . | OR/35-49 |
| 51 | . | 34 and 50 |
